# Coexistence of immunoglobulin G4-related kidney disease and acute hematogenous disseminated pulmonary tuberculosis: a case report

**DOI:** 10.3389/fimmu.2024.1493754

**Published:** 2025-01-09

**Authors:** Fangfang Zhou, Hanqing Chu, Youjun Xu, Yena Zhang, Kuibi Tan, Jinxia Ge, Ningjun Shao, Qun Luo

**Affiliations:** ^1^ Department of Nephrology, Ningbo No.2 Hospital, Ningbo, Zhejiang, China; ^2^ Department of Nephrology, Yuyao People’s Hospital, Ningbo, Zhejiang, China; ^3^ Department of Pulmonary Tuberculosis, Ningbo No.2 Hospital, Ningbo, Zhejiang, China

**Keywords:** IgG4-related kidney disease, tuberculosis, case report, acute kidney injury, free light chain (FLC)

## Abstract

**Background:**

Immunoglobulin G4-related disease (IgG4-RD) is an immune-mediated fibrous inflammatory disease. Recently, an association between IgG4-RD and tuberculosis (TB) has been reported.

**Case summary:**

We report a 56-year-old man complaining of a cough and poor appetite for 2 months and oliguria for 1 day. The patient was diagnosed with TB due to a manifestation of lymphatic TB and the radiological alterations of acute miliary pulmonary TB. He also presented with greatly elevated serum creatinine, non-albumin proteinuria, immunoglobulin subgroup IgG4, and immunoglobulin free light chain (FLC) levels. A diagnosis of IgG4-RKD was suggested by a renal biopsy. We then administered the patient glucocorticoid and anti-TB treatment for 4 months. The patient’s renal function was completely restored and the manifestations of TB were alleviated.

**Conclusion:**

The necessity and complexity of differential diagnosis in patients with coexisting IgG4-RD and TB remains challenging. Early recognition and timely treatment are important for averting its progression. Long-term monitoring is required to assess for recurrence of IgG4-RD and TB activity.

## 1 Introduction

Immunoglobulin G4-related disease (IgG4-RD) is an immune-mediated fibrous inflammatory disease, occurring in middle and old age, and the ratio of men to women who are diagnosed with the disease is approximately 8:3 (1). The main histopathological manifestations included lymphoplasmacytic cell infiltration dominated by immunoglobulin subtype 4 (IgG4)-positive plasma cells and specific tissue fibrosis such as retroperitoneal fibrosis and orbital pseudotumor. Accordingly, the proliferative and fibrotic phenotypes of IgG4-RD have been proposed. It is usually difficult to distinguish between them because the two phenotypes can be present concurrently.

Recently, an association between IgG4-RD and tuberculosis (TB) has been reported. Although the mechanism between these two diseases is still unknown, some common immune responses may be shared by both TB and IgG4-RD. According to recent literature, the manifestations of TB and IgG4-RD can occur successively or simultaneously, with various organs involved. Qing et al. reported that 36.2% (17/47) of the patients with IgG4-RD in their single-center study presented with concurrent TB disease or latent TB infection (2). Moreover, most of the TB-related cases in which IgG4-RD was diagnosed were predominantly the fibrotic phenotype, such as fibrosis or sclerosis in the glandular, periorbital, retroperitoneal tissue, or lymph nodes. Herein, we report a rare case with a proliferative phenotype of IgG4-RD coexisting with acute hematogenous disseminated pulmonary TB. The patient was successfully treated with prednisone and anti-tuberculosis drugs for 4 months.

## 2 Case presentation

**Figure 1:**
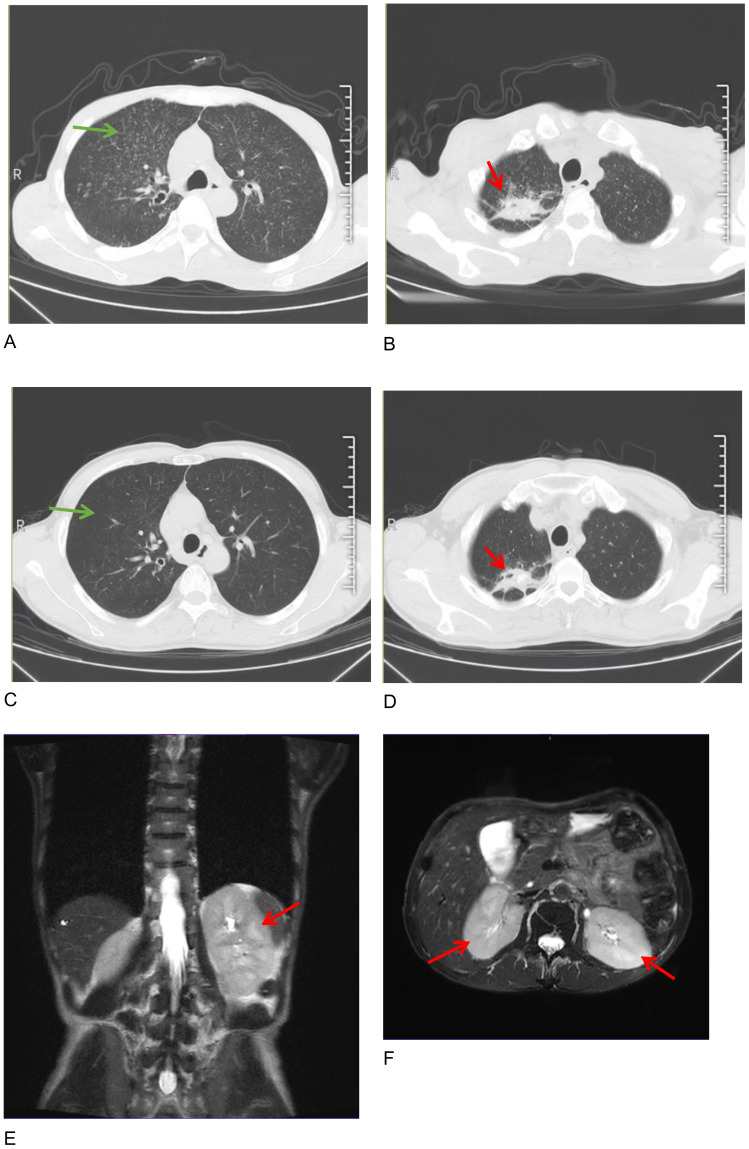
Chest computed tomography images on admission **(A, B)** and half a year after therapy **(C, D)**, showing that diffuse miliary changes in both lungs (green arrows in **A, C**) were almost absorbed and the polymorphic lesions in the upper lobe of the right lung were controlled (red arrows in **B, D**). Axial and coronal images of a renal magnetic resonance imaging (MRI) scan showing the enlargement of the two kidneys on admission (**E, F**, red arrow).

A 56-year-old man presented with a cough, a poor appetite, and oliguria. Furthermore, 2 months before admission, the patient had a recurrent cough, intermittent fever at night, and touchable bilateral cervical lumps. He had been diagnosed with secondary pulmonary TB 2 and cervical lymph node TB according to the results of a cervical lymph node puncture in the local hospital and received an HREZ regimen [isoniazid (H) 0.1g qd, rifampin (R) 0.15g qd, ethambutol (E) 0.25g qd, and pyrazinamide (Z) 0.25g tid]. During the treatment (approximately 1 month), the patient’s symptoms of cough and fever were relieved but developed a poor appetite. The anti-TB regimen was then changed to isoniazid 0.3 qd, rifapentine 0.45 q72h, ethambutol 0.75 qod, and moxifloxacin 0.4 qd, considering the side effects of hepatoxicity and nephrotoxicity. One day before admission (5 days after starting the changed anti-TB regimen), he had oliguria (urinary output <400 ml/d) and was admitted to the emergency room of our hospital. Upon admission (29^th^ Mar), the patient exhibited acute kidney injury (AKI) [serum creatinine (sCr) of 800μmol/L, while sCr was 105μmol/L on 8 March], hyperglobulinemia (54.7g/L) with hypoproteinemia (31.5g/L), hypokalemia (3.02mmol/L), and anemia (83g/L) with thrombocytopenia (82 *10^9/L). Arterial blood gas analysis showed metabolic acidosis (pH 7.31, BE -13.1). Chest and abdominal computed tomography (CT) scans showed diffuse miliary lesions in both lungs (**Figure 1A**, green arrow), old lesions in the upper lobe of the right lung that may have been due to TB (**Figure 1B**, red arrow), mediastinal lymph node calcification, and enlargement of both kidneys. According to his history of TB, the patient was transferred to the Department of Pulmonary Tuberculosis, and the Department of Nephrology was consulted.

**Figure 2:**
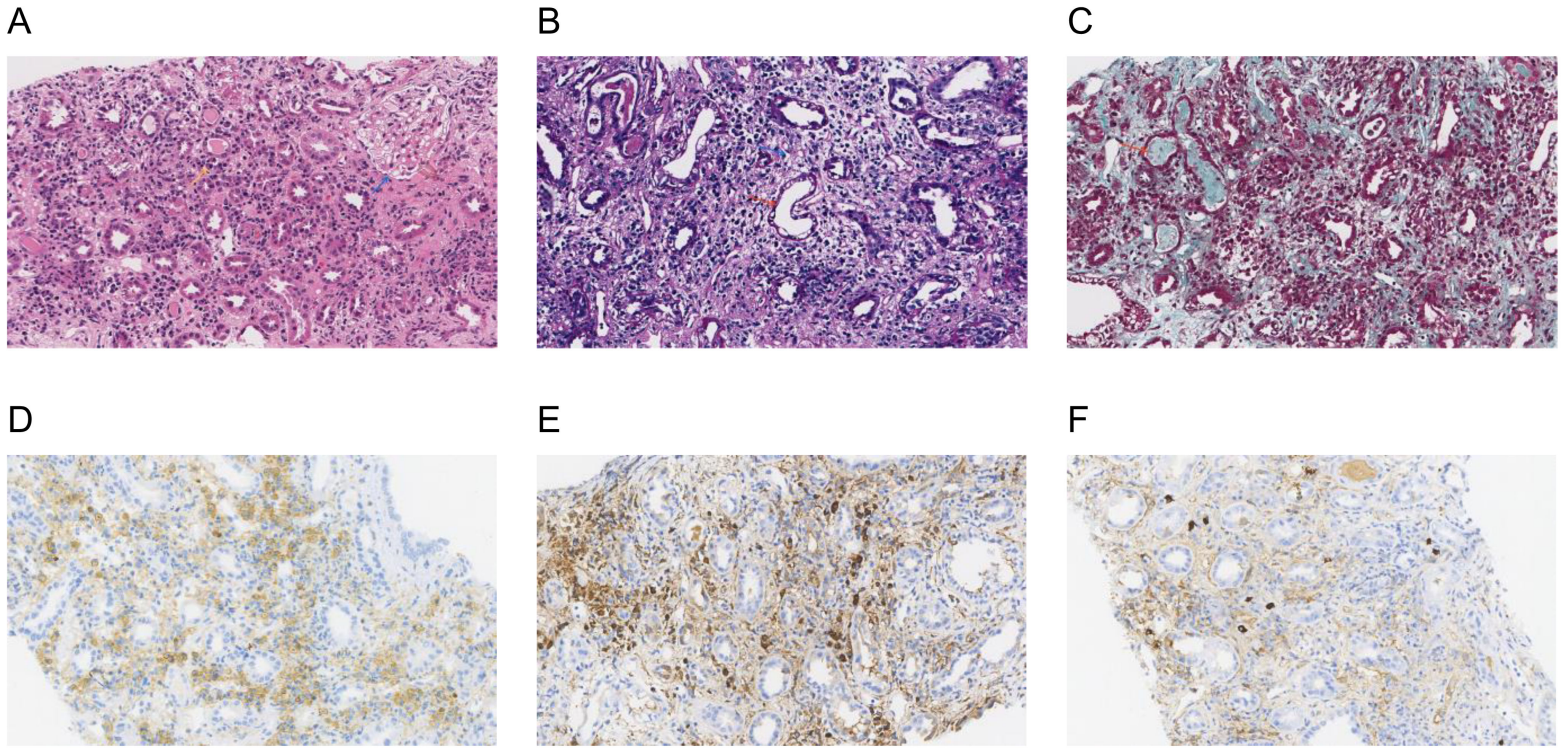
Renal biopsy: a histological section of the kidney parenchyma was consistent with the diagnosis of acute interstitial nephritis. **(A)** Hematoxylin and eosin staining (×200) showed normal glomeruli (blue arrow) and tubulointerstitial lymphoplasmacytic infiltration (yellow arrow); **(B)** Periodic acid-Schiff staining (×200) showed dilated lumen in some renal tubules, the disappearance of brush edges (red arrow), and interstitial edema (blue arrow); **(C)** Masson trichrome staining (×200) showed a small number of proteins and granular tubular types (red arrow). Immunohistochemical staining (×200): **(D)** CD38 staining was diffusely positive for CD38-positive plasma cells; **(E, F)** IgG and IgG4 staining showed >10 IgG4-positive plasma cells per high powered field and the ratio of IgG4/IgG plasma cells was >40%, consistent with a diagnosis of IgG4-related kidney disease.

Further laboratory tests were performed, showing IgG 32.82g/L, IgM 2.34g/L, IgG4 3.51g/L, and complement C1q 455.56mg/L, with normal levels of IgA and complements C3 and C4. Immunoglobulin kappa free light chain (FLC) levels were >250.00mg/L in the serum and >250.00mg/L in the urine, while the lambda levels were 180.39mg/L in the serum and >250.00mg/L in the urine. Both serum and urine immunofixation electrophoresis (IFE) were negative for FLCs and monoclonal proteins. Urine analysis showed protein 1+ and glucose 3+ in the routine urine test; the patient’s urine protein-to-creatinine ratio (PCR) was 2549.8 mg/g, urine albumin-to-creatinine ratio (ACR) was 174.2 mg/g, 24-hour urine microalbumin was 101.3 mg, and 24-hour urine total protein was 1938.8mg, indicating the protein in the urine was non-albumin. Other laboratory tests showed hypocalcemia (1.84mmol/L), hypomagnesemia (0.63mmol/L), hyperphosphatemia (2.13mmol/L), elevated erythrocyte sedimentation rate (ESR) (41.0mm/h), C-reactive protein (CRP) (31.64mg/L), and some tumor markers (glycosyl antigen 125: 32.70U/mL, glycosyl antigen 199: 44.67U/mL). Autoantibody and vasculitis-related antibodies tested negative. Sputum smears for tuberculosis (three in total) were all negative. The T-SPOT.TB test was positive (++). An ultrasound revealed multiple swollen lymph nodes (the size of the biggest ones were 44*15mm on the right and 55*18mm on the left) in both neck regions, which were suspected to be lymphatic tuberculosis, partially liquefied. The size of the left kidney was 116 × 54mm, and the size of the right kidney was 119 × 50mm. The shape of both kidneys was full, and the echo was reduced and uneven. A renal magnetic resonance imaging (MRI) scan also showed enlargement of the two kidneys (**Figures 1E, F**, red arrow). A cervical lymph node puncture was then performed with the results showing granulomatous lesions with necrosis: acid-fast staining (+ weak), silver hexamide (-), and fungal periodic acid-Schiff staining with diastase(-), without IgG4 positive cells. A *Mycobacterium tuberculosis* (MTB) rpoB gene and mutation test of the cervical lymph node (Xpert MTB) was positive (+). Finally, a renal biopsy suggested acute tubulointerstitial nephritis (**Figures 2A–C**) with a large number of infiltrated plasma cells and multifocal interstitial infiltration of CD38-, IgG-, and IgG4-positive cells, with an IgG4-positive plasma cell level >10/HP, and the ratio of IgG4/IgG plasma cells was >40% (**Figures 2D–F**).

**Figure 3:**
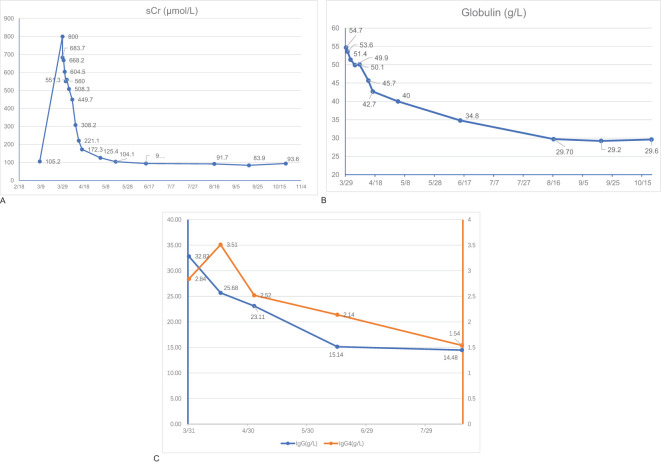
Laboratory findings. Serum levels of serum creatinine (sCR) **(A)**, globulin **(B)**, Immunoglobulin G (IgG), and Immunoglobulin G4 (IgG4) **(C)** of the patient during the follow-up.

Moxifloxacin (0.4 qd), isoniazid (0.3 qd), linezolid(0.6 qd), and rifapentine(0.45 q72h) were administered for anti-tuberculosis treatment. Prednisone (30mg/d for the first month, and tapered to 5mg/2 weeks) was administered when IgG4-RD was diagnosed (6 April). The patient experienced considerable improvement in his symptoms and laboratory and imaging examinations after 4 months of treatment [16 August: sCr 91.70μmol/L; globulin 29.7g/L, IgG 14.48g/L, and IgG4 1.54g/L (**Figures 3A–C**); urine PCR: 624.1mg/g, ACR:28.8mg/g]. FLCs were decreased to 154.35mg/L in the serum and 362.32mg/L in the urine for kappa light chains and 39.68mg/L in the serum and 948.06mg/L in the urine for the lambda light chains. Serum albumin, electrolytes, hemoglobin, platelets, etc. had all returned to the normal range.

The patient has been followed up for half a year. He is currently administered prednisone 5mg/d for maintenance. His kidney function is stable (sCr 93.60μmol/L, with urine PCR: 214.0mg/g, ACR:5.5mg/g). Moxifloxacin and linezolid were stopped because both chest CT (**Figures 1C, D**) and an ultrasound of the lymph nodes (the size of the biggest ones was reduced to 31*7mm on the right and 40*9mm on the left) showed partial foci absorption of the TB.

## 3 Discussion

The present case showed a simultaneous existence of TB and IgG4-RD. The patient presented with acute disseminated pulmonary TB and AKI which was diagnosed as IgG4-RKD by a renal biopsy. It is a relatively rare case because the patient was diagnosed with both IgG4-RD in the proliferative phase and acute hematogenous disseminated pulmonary TB. The patient’s TB and renal function symptoms improved gradually when glucocorticoid and anti-TB therapies were initiated.

China has a high TB burden, ranking third in the estimated number of TB cases. Patients with TB have been noted to have a relatively high prevalence of IgG4-RD either after the development of TB or simultaneously. However, the clinical manifestations and lung imaging of IgG4-RLD are varied and lack specificity. The initial radiological manifestations of IgG4-RLD are similar to those of pulmonary TB and could even mimic TB (3). Hence, IgG4-RLD is often misdiagnosed as TB, pneumonia, lung tumor, interstitial pneumonia, etc. It is also difficult to make an early diagnosis when TB and IgG4-RD coexist (4). A histopathological examination is the key to a definitive diagnosis. Unfortunately, a lung biopsy was not carried out because the patient refused. However, the patient had the symptoms of cough and fever at night and was positive in TB-related tests such as T-SPOT, Xpert MTB, and acid-fast staining of the cervical lymph nodes. The manifestation of lymphatic TB and the radiological alterations of acute miliary pulmonary TB also supported the diagnosis of TB.

The patient also presented with an AKI, the etiology of which needed to be differentiated. Postrenal obstruction was first ruled out by imaging. The presence of nausea and poor appetite before his admission suggested the possibility of prerenal AKI. The medication history of nephrotoxic anti-TB drugs such as rifampin and pyrazinamide might also have contributed to the development of an AKI. It has been reported that more than half of AKI episodes happen in the first 2 months of the anti-TB treatment (5). In this case, the patient presented a poor appetite after anti-TB treatment as well as hypoalbuminemia, anemia, and thrombocytopenia, which are the most common laboratory findings of an anti-tuberculosis drug-related AKI. The differential diagnosis of AKI was finally determined by a renal biopsy. It showed acute tubulointerstitial nephritis with a large number of infiltrated plasma cells and multifocal interstitial infiltration of CD38-, IgG- and IgG4-positive cells, with an IgG4-positive plasma cell level of >10/HP, and an IgG4/IgG plasma cell ratio of >40%, supporting the diagnosis of IgG4-RKD (6). No storiform fibrosis was observed in the renal histopathology, suggesting the patient was in the proliferative phase of IgG4-RD (7). Meanwhile, the elevated serum IgG4 concentration and radiographic manifestation of diffuse enlargement of the kidney also confirmed the diagnosis of IgG4-RD.

A previous study reported kappa and lambda FLC levels were higher in IgG4-RKD patients (8). They are considered biomarkers associated with IgG4-RD disease activity and renal involvement. IgG4-RD can even mimic monoclonal gammopathy, highlighting the hyperactivation of B cells. In this case, the patient also presented with non-albumin proteinuria, increased FLC and IgG4 levels, an elevated serum kappa/lambda ratio, and hyperglobulinemia, but no monoclonal proteins were observed in IFE. Considering the tendency of transformation from IgG4-RD into lymphoid neoplasms, it is necessary to exclude plasmacytosis and lymphoid neoplasms in patients with IgG4-RD and an abnormal kappa/lambda ratio. We should also pay attention to the risk of lymphoproliferative diseases during follow-up.

Few cases have been reported linking TB and IgG4-RD. The mechanism between these two diseases remains unclear. One hypothesis was that MTB activates the Th2 response, which in turn activates the production of IgG4 antibodies and IgG4-RD is developed (9). Current research suggests that IgG4-RD might share some common immune responses with TB infection during development (2). Both abnormal adaptive and innate immune responses play important roles in the pathogenesis of IgG4-RD (10). Single-cell RNA sequencing revealed that the number of B cells, plasma cells, and CD4+ T cells increased in IgG4-RD retroperitoneal tissues (11). It has been found that CD4+ cytotoxic T lymphocytes release interleukin-1β, interferon-gamma (IFN-γ), and transforming growth factor (TGF-β1), participating in the pathogenesis of IgG4-RD. It is also demonstrated that B cell activation and differentiation into IgG4-secreting plasma cells participate in the development of IgG4-RD pathogenesis (12). As for TB, it is not only a bacterial infectious disease, but also an immune disease. In the acute phase, CD4+ T cells and INF-γ dominate, and a humoral immune response mediated by B cells is also involved (2). However, how these common immune mechanisms are linked and function still needs to be explored.

IgG4-RD can progress to permanent organ dysfunction. This is mainly dependent on the duration of the proliferative phase before recognition and treatment. Hence, early detection following timely treatment is important for improving organ function and preventing progression to prominent tissue fibrosis. To date, corticosteroids remain the cornerstone of the treatment of IgG4-RD and are recognized as first-line agents for the induction and maintenance phases of the disease (13). Recently, the evidence for immunosuppressors such as mycophenolate, azathioprine, methotrexate, and rituximab for the treatment of IgG4-RD has been increasing (14, 15). The results showed that immunosuppressive therapy could improve the efficacy of treatment and can be used in combination with corticosteroids in the initial treatment phase or during the process of corticosteroid reduction. In this case, we used glucocorticoid monotherapy once IgG4-RKD was diagnosed. Due to the patient’s proliferative phenotype of IgG4-RD, the patient’s symptoms were expected to respond well to glucocorticoid monotherapy (6). Anti-TB drugs with low nephrotoxicity such as moxifloxacin, isoniazid, linezolid, and rifapentine were used for the anti-TB therapy. The patient achieved complete remission of IgG4-RKD and partial remission of TB. It is important to note that the use of corticosteroids and/or immunosuppressors inevitably increases the risk of opportunistic infections, especially when combined with TB infection. It is necessary to closely monitor the indicators of opportunistic infection and TB reactivity during follow-up. IgG4-RKD should also be monitored long-term considering its recurrent nature. Moreover, high-quality clinical trials for IgG4-RD are needed, especially in conjunction with infectious diseases such as TB.

## 4 Conclusion

The necessity and complexity of a differential diagnosis in patients with coexisting IgG4-RD and TB remains challenging, so a histopathological examination is important. Early recognition and timely treatment are important for averting its progression. Long-term monitoring is required to assess for the recurrence of IgG4-RD and TB reactivity.

## Data Availability

The original contributions presented in the study are included in the article/supplementary material. Further inquiries can be directed to the corresponding author.
